# Development of a customized mask retainer for improving the fit performance of surgical masks

**DOI:** 10.1371/journal.pone.0278889

**Published:** 2022-12-09

**Authors:** Yuanyuan Pan, Qi Xi, Jiali Meng, Xi Chen, Guofeng Wu

**Affiliations:** 1 Department of Prosthodontics, Nanjing Stomatological Hospital, Medical School of Nanjing University, Nanjing, China; 2 Xuanwu Stomatological Hospital, Nanjing, China; 3 Digital Engineering Center of Stomatology, Nanjing Stomatological Hospital, Medical School of Nanjing University, Nanjing, China; 4 Digital Stomatology Center, the Affiliated Hospital of Shaanxi University of Chinese Medicine, Xianyang, China; Universiti Sains Malaysia, MALAYSIA

## Abstract

This study introduces a customized mask retainer to improve the fit performance of surgical masks using various advanced digital techniques. The participant’s 3D face scans with and without a surgical mask were taken by using a smartphone. The mask retainer was designed using the 3D face scan data based on the facial anthropometric landmarks. The fitting was inspected and adjusted using the masked face scan data. The retainer was fabricated using a 3D printer. The effectiveness of the retainer on the augmentation of the fit of the surgical mask was tested according to the Chinese Standard (GB 19083–2010). A questionnaire was used to assess the effect of wearing surgical masks with and without retainers and N95 respirators on subjective perception of discomfort. The effectiveness test of the retainer on the augmentation of the fit performance showed a better than 25-fold increase in the overall fit factor, meeting the fit requirement for KN95 respirators in China. The subjective perception of discomfort of wearing N95 was significantly greater than surgical mask with and without retainers. The fit factor results indicated that by using the retainer, the overall fit factors and that of each exercise significantly increased compared to that of the group with the surgical mask alone. And compared with N95, the surgical mask with the retainer significant improved comfort. The surgical mask with the retainer can provide an alternative of personal protective equipment for healthcare workers.

## 1. Introduction

Healthcare workers face a higher risk of infection than ordinary people [1]. Infectious aerosols generated by patient coughing, sneezing, or interventions such as endotracheal intubation and dental treatments using high-speed handpieces and ultrasonic instruments may contain various pathogenic microorganisms [2–5], including the COVID-19 epidemic [6]. Disposable surgical masks, together with N95 respirators, gloves, gowns, and face shields, are recommended personal protective equipment (PPE) against nosocomial infections in clinics [7]. The WHO recommends that healthcare workers wear medical masks or a respirator when entering rooms of patients with suspected or confirmed COVID-19. N95 respirators are recommended for medical procedures that generate fine-particle aerosols [8]. The COVID-19 pandemic has exacerbated problems related to PPE fitness. Most PPE designs are based on anthropometric data from European and American average people and are clearly not suitable for everyone. Surgical masks are not particularly suitable for most people, and this mismatch can lead to constant adjustments and increase the risk of unnecessary infections [9, 10]. Furthermore, particulate respirators do not provide adequate protection for people with beards, potentially providing a false sense of security [11].

Surgical masks have a lower respiratory infection protection capacity than N95 respirators (both in laboratory and clinical settings), primarily because the former have a lower leak-tight fit on the user’s face [12], leading to the leakage of contaminated air from and into the breathing zone [13–15]. The overall filtration efficiency of the mask is affected by improper wearing or poor facial fit [16]. Nevertheless, a meta-analysis showed that surgical mask wear was remarkably higher than N95 respirators. Wearing N95 respirators for a long time was likely to cause discomfort such as headaches and breathing difficulties [17]. Global healthcare workers were also facing higher risks of infection in their efforts to protect the greater community and are exposed to hazards such as psychological distress and fatigue [18, 19]. Therefore, enhancing the sealing of surgical masks would be a beneficial solution.

Several studies have employed auxiliary devices to improve the fit performance of facepieces enabling better border seal and adaptation [20–24]. The 3D facial scanning technique was recently used to collect facial anthropometric data for the design and 3D printing of customized respirator accessories with better adaptation to the individual’s face contour [22–24]. However, methods to improve the leak-tight fit of commercially available surgical masks are seldom reported. This study aimed to introduce a digital PPE solution for healthcare workers. Common digital equipment such as smartphones and 3D printers, has been successfully applied to improve personal protection significantly.

## 2. Materials and methods

### 2.1. Subjects

The study was conducted at the Nanjing Stomatological Hospital, Medical School of Nanjing University. Ten healthy subjects (dentists, five males, and five females, age range is 35–45 years) were enrolled. The characteristics of the participants are shown in Table 1. Every participant was tested three times at the same time of day on three different days, wearing one of three types of facemasks. This study was approved and supervised by the Ethics Committee of Nanjing Stomatological Hospital, Medical School of Nanjing University. The individual in this manuscript has given written informed consent (as outlined in PLOS consent form) to publish these case details.

**Table 1:** Characteristics of subjects.

Characteristic	Male			Female		
	Average	SD	Range	Average	SD	Range
**Age(years)**	40.2	3.3	35–45	40	4.1	35–45
**Weight(kg)**	76.4	6.2	69–85	55.4	7.8	45–65
**Height(cm)**	175.6	7.3	166–185	159.6	7.2	150–169

The table describes the average, standard deviation and range of age, weight and height of the subjects.

### 2.2. Digital workflow of customized mask retainer

**Fig 1:**
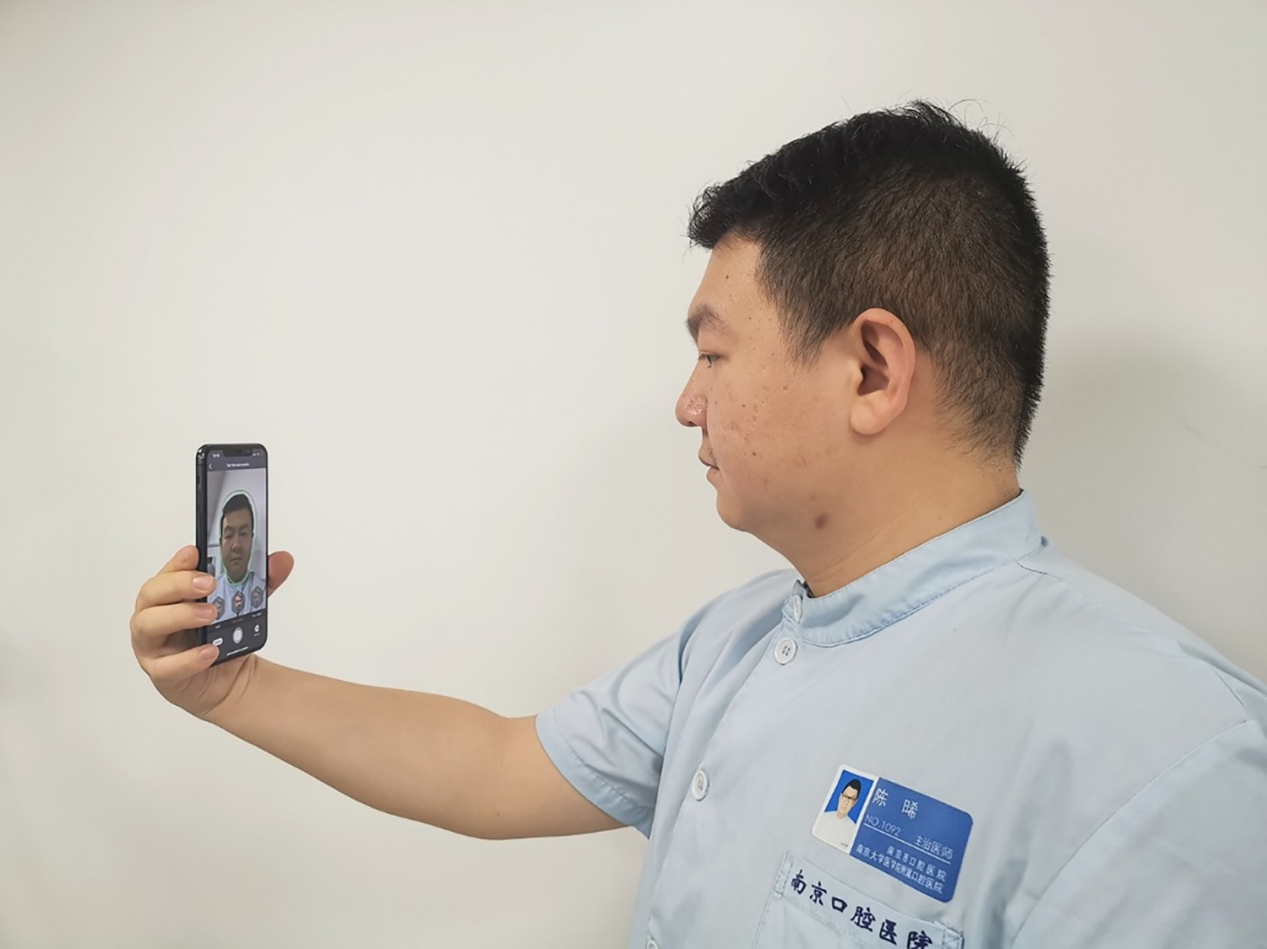
3D face scan by using scanning APP. Participant taking 3D face scan with scanning APP installed on a smartphone. Whole process cost less than 1 minute.

3D face scan data (with and without a surgical mask) were captured using a 3D facial scanning app (Dental Pro; Bellus3D) installed on a smartphone (iPhone 11 Pro; Apple Inc.), according to the app’s instructions (Fig 1).

**Fig 2:**
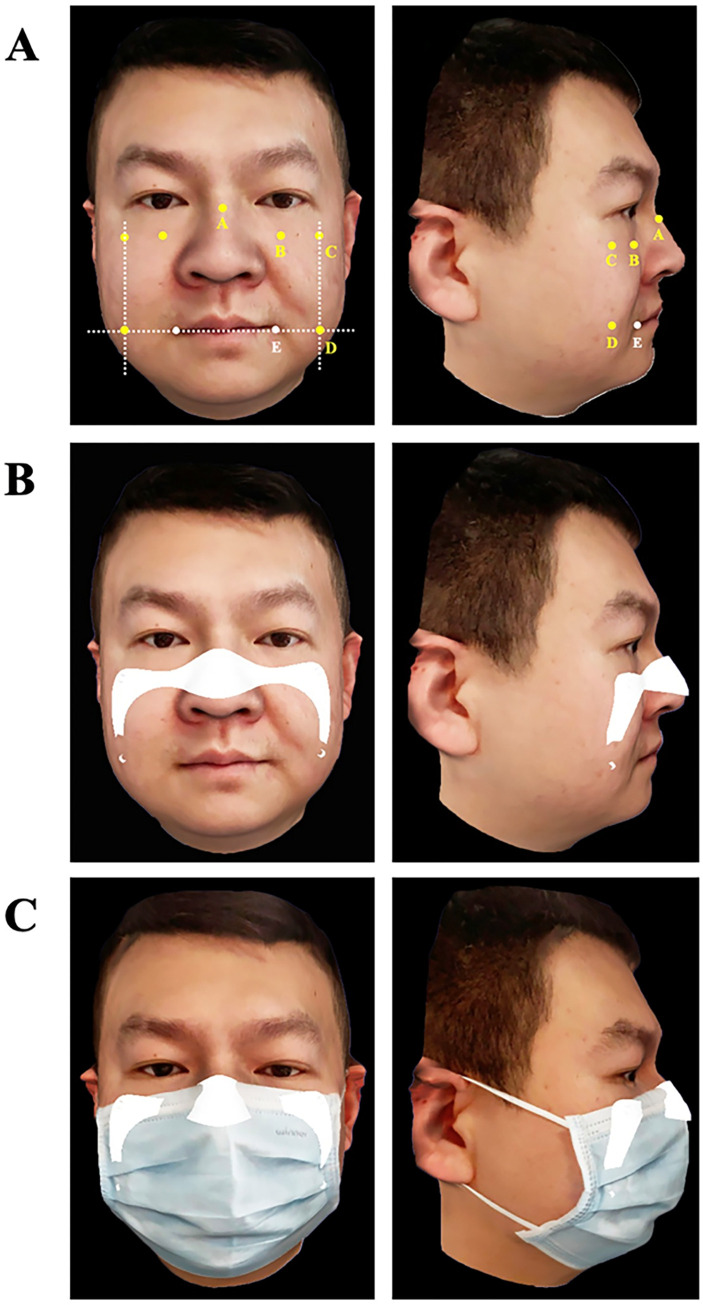
3D face scans acquired with face scanning APP and CAD of retainer. Panel A shows anatomical facial landmarks used to determine border of retainer: A, rhinion; B, margo infraorbitalis; C, processus temporalis ossis zygomatici; D, vertical line at angulus oris plane; E, angulus oris. Panel B shows CAD of retainer merged on 3D face scan. Panel C shows CAD of retainer merged on 3D face scan with mask. CAD, computer aided design.

The scanned data in standard tessellation language (STL) format was imported into CAD software (Magics v24.0; Materialize). The primary contour line of the retainer was extracted based on constant anatomical facial landmarks: the superior border retainer was placed at the rhinion (the anterior tip at the end of the suture of the nasal bones) and the margin infraorbitalis plane to the processus temporalis ossis zygomatici, which were extended to the angulus oris plane (Fig 2A). Triangular patches of the main contour were extracted and solidified to a thickness of 1.2 mm, with supports for elastic straps provided at the upper and lower borders of the flanks (Fig 2B). The design of the retainer was checked on a facial scan with a surgical mask for contour scope and adaptation so that the retainer did not exceed the border of the mask (Fig 2C).

**Fig 3:**
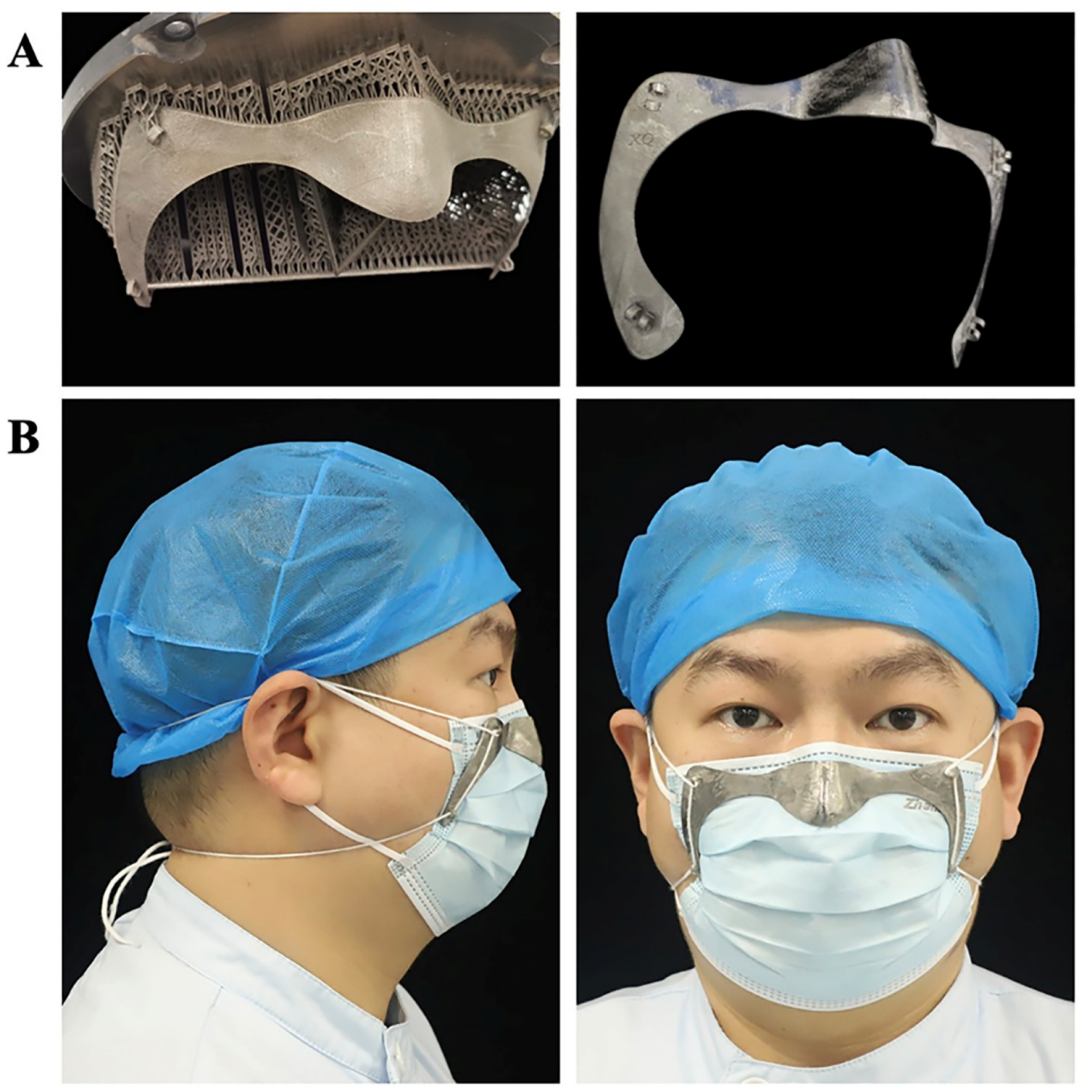
Surgical mask retainer. Panel A shows fabrication of the retainer by using 3D printing technology before and after support was removed. Panel B shows participant wearing surgical mask supplemented by the retainer.

The CAD of the retainer was exported into the STL file format, printed using a metal printer for Titanium (Ti150; Profeta Intelligent Technology Co., Ltd.) in Ti6-Al4-V alloy, and finished by manually removing the supports and polishing (Fig 3).

### 2.3. Fit factor test

**Fig 4:**
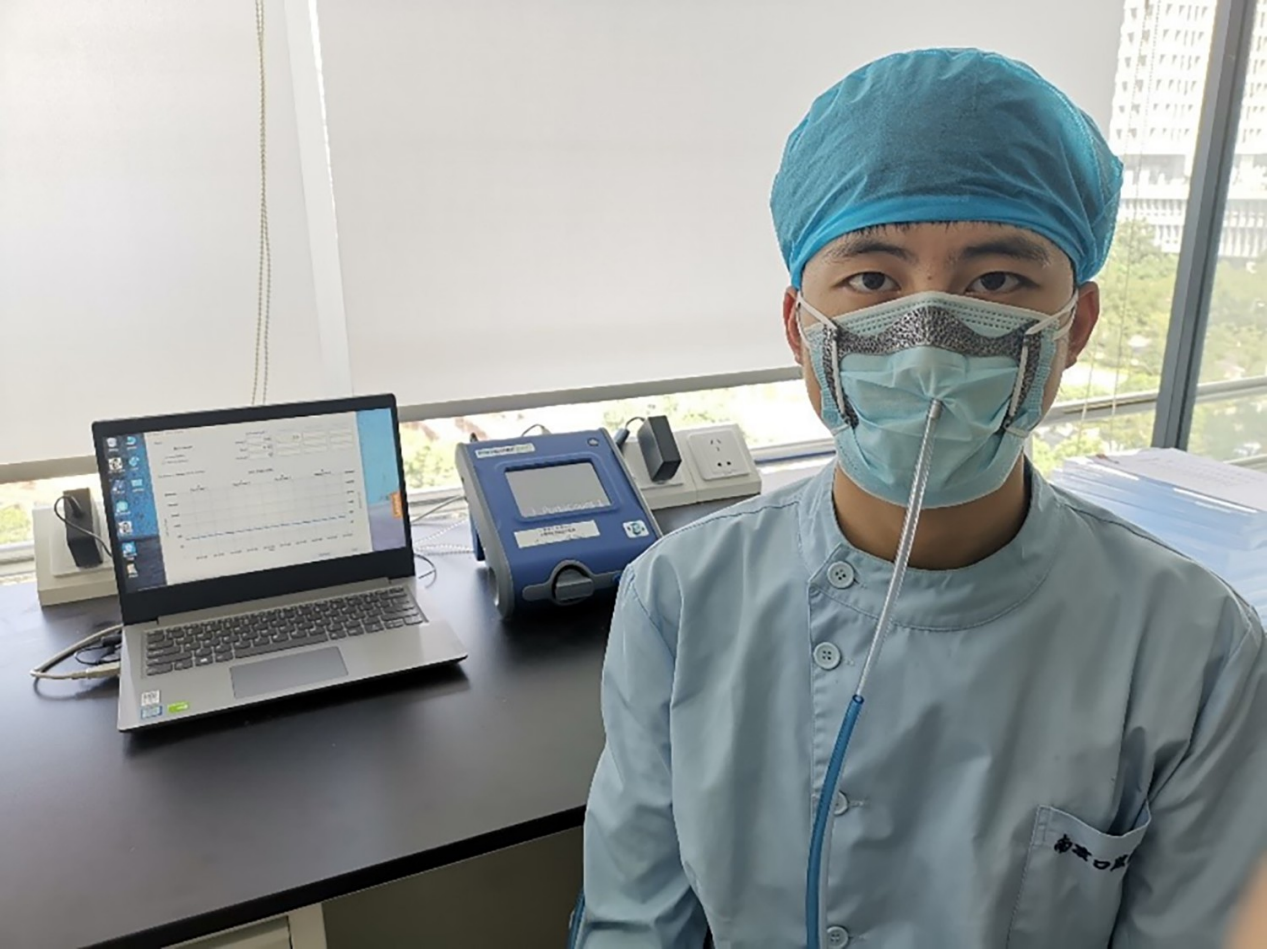
Participant taking fit factor test. Shown is fit testing situation for participant wearing surgical mask supplemented by retainer. The subjects’ favorite pattern is added to the upper left corner of the retainer.

FF tests were performed to validate the actual effect of the retainer on the seal of the surgical mask. A retainer was fabricated for each participant using the method described above. A commercial elastic strap was attached to the retainer using a length-adjusting clip. The retainer was placed on the outside of the surgical mask and adjusted to achieve ideal adaptation and comfort. The fit factor (FF) of the surgical mask (Surgical Mask; Winner Medical Group Inc., China) without a retainer (M group, n = 10) and with a retainer (MR group, n = 10) were tested using a respirator fit tester (Portacount Pro+; TSI) according to the Chinese Standard “Technical requirements for protective surgical mask for medical use” (GB 19083–2010). The FF was calculated as the ratio of the concentration of air particles in the environment and in the inhaled air under the mask during a cycle of six exercises (each for 1 min): initial normal breathing (iNB), deep breathing (DB), head movement from side to side (Head L/R), head movement up and down (Head U/D), talking (Talk), and final normal breathing (fNB) (Fig 4).

### 2.4. Subjective perceived discomfort and study design

**Fig 5:**
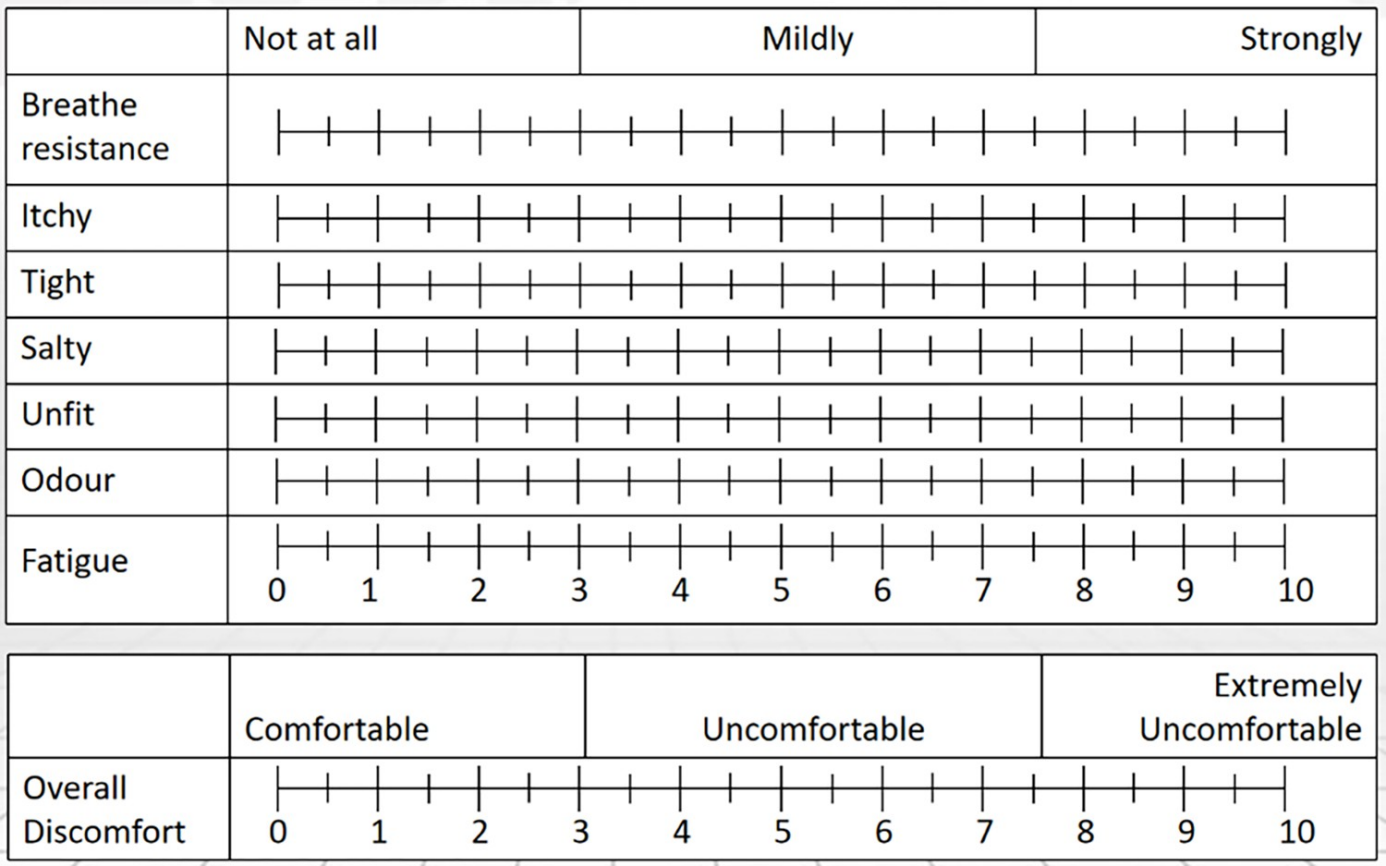
Rating scale of subjective perceived discomfort. Questionnaire of quantifying eight domains of comfort/discomfort of wearing a mask.

This study referred to a published questionnaire published by [25] to quantify the following eight domains of comfort/discomfort of wearing a mask: breathing resistance, itchiness, tightness, saltiness, feeling unfit, odor, fatigue, and overall discomfort. The rating scale is shown in Fig 5. The study design is as follows: participant wore a mask and rested on a chair for 30 min, then walked on a treadmill at 3.2 km/h for 20 min and rested for 10 min; walked for 10 min at 4.8 km/h and rested for 10 min; walked for 10 min at 6.4 km/h and rested for 10 min again. The participants were asked at 60, 80 and 100 min of the intermittent exercise how they perceived the comfort in the questionnaire.

### 2.5. Statistical analysis

The questionnaire for each type of mask and the fit factor tests were repeated three times per participant. The medians of individual and overall FF were used to compare the differences between the groups using the Wilcoxon matched-pairs signed-ranks test using software (IBM SPSS Statistics v22.0; IBM Corp). Data of subjective rating were analyzed using GraphPad Prism 8 (GraphPad Software Inc., California, USA). Comparisons were made using Kruskal-Wallis test and Dunn’s post hoc test (a = 0.05).

## 3. Results

### 3.1. Fit factor test

The results listed in Table 2 indicate significant increases in FF for the MR group in each exercise and a more than 25-fold increase in the overall FF (M:6; MR:154), which satisfied the requirement for the KN95 respirator as detailed in the Chinese Standard.

**Table 2:** Scores of FF of surgical mask with and without retainer.

Group	Exercises						Overall Score
	iNB	DB	Head L/R	Head U/D	Talk	fNB	
M	10	7	4	7	6	8	6
MR	188	136	123	144	84	165	154
**Z Value**	-2.805	-2.805	-2.807	-2.807	-2.807	-2.805	-2.805
**P**	.005	.005	.005	.005	.005	.005	.005

M group: surgical masks without a retainer. MR, group of surgical masks with retainers. iNB, initial normal breathing. DB, deep breathing. Head L/R, head movement from side to side. Head U/D, head movement up and down. fNB, final normal breathing.

### 3.2. Subjective perceived discomfort

The subjective ratings of discomfort in eight domains for the three types of masks are shown in Table 3. The degree of discomfort of all items of N95 respirators was significantly higher than that of the other two types of masks. The breathing resistance of surgical masks with retainers was slightly higher than that of surgical masks, but there was no significant difference in other aspects.

**Table 3:** Subjective perceived discomfort.

	M	MR	N95	ANOVA	M vs MR	M vs N95	MR vs N95
**Breathe resistance**	4.3±1.4	5.9±1.5	7.9±2.0	<0.0001	0.0093	<0.0001	0.0031
**Itchy**	3.4±2.1	3.2±1.8	5.1±2.3	0.0066	>0.9999	0.0217	0.0155
**Tight**	3.4±1.9	3.1±2.0	7.1±1.7	<0.0001	>0.9999	<0.0001	<0.0001
**Salty**	2.0±1.2	1.9±0.9	5.4±1.9	<0.0001	>0.9999	<0.0001	<0.0001
**Unfit**	3.1±2.2	2.4±2.0	5.8±1.9	<0.0001	0.5823	0.0004	<0.0001
**Odor**	2.9±1.7	3.1±1.9	5.5±1.3	<0.0001	>0.9999	<0.0001	<0.0001
**Fatigue**	4.0±1.8	4.4±1.7	6.0±2.1	0.0009	>0.9999	0.001	0.0189
**Overall discomfort**	5.0±1.8	5.4±2.0	6.9±2.1	0.0031	>0.9999	0.0034	0.0401

Results of the questionnaire quantitating eight domains of comfort/discomfort of wearing a surgical mask (M) and a surgical mask with mask retainer (MR) compared to a N95 respirator (N95) on a scale from 0 (comfortable) to 10 (extremely uncomfortable) depicted as mean ± standard deviation.

## 4. Discussion

The customized mask retainer in this study was designed and processed according to the 3D face scan data of participants, which not only ensures the comfort and effectiveness of masks but provides a personalized PPE choice for people in need. Primary results of FF tests of the surgical mask supplemented with a retainer indicated a significant improvement as compared to the surgical mask alone group. The fit test is not mandatory for surgical masks according to the Chinese Standard, and the present study revealed a comparatively low FF value for the surgical mask alone group. Using the retainer, FF increased to >100, which satisfies the Chinese Standard “Technical requirements for protective surgical mask for medical use” (GB 19083–2010) for respirators, such as N95. The leakage of the facial seal area of surgical masks is the main factor in measuring whether the masks can effectively protect the wearer. Compared with the filtering efficiency of the material used in the masks, the sealing of the surgical masks is much more important in reducing the total inward leakage and protecting the wearer [26]. However, their sealing ability is often poor, which is attributed to insufficient adaptation to the individual’s face contour, especially during talking, and head and jaw movements. Surgical masks must not only be made of highly filtered, low-resistance material but is suitable for the wearer to provide adequate respiratory protection. Even minor anatomical changes can have a significant impact on fitness [27]. A recent study found that by covering medical masks with rubber bands, nylon hosiery, or other measures to enhance the fit between medical masks and the wearer’s face, the fitted filtration efficiencies of masks could be improved, indicating the importance of mask fit in maximizing filtering efficiency. However, not all modifications are comfortable and practical for long-term use [28]. The customized mask retainer used in this study was more suitable for participants’ facial features and conducive to long-term use.

Other important considerations for PPE are factors such as comfort, breathing resistance, sources of supply and cost [29, 30]. The results of the subjective discomfort questionnaire showed that wearing a N95 respirator was more uncomfortable than wearing a surgical mask with and without retainer. The surgical mask with the retainer not only can enhance the sealing of masks, but also benefit the physical and mental health of the wearer. A study reported that N95 has significant negative impacts on cardiopulmonary exercise capacity, highly impairing physical function, and quality of life [31]. Furthermore, PPE use may cause adverse skin reactions. Hu et al. found that approximately 95% of participants (healthcare workers) who regularly wore N95 respirators experienced adverse skin reactions such as, nasal bridge scarring, facial itching, skin damage, dry skin, and rash. However, healthcare workers who used surgical masks did not report any adverse skin reaction [32]. The comfort of surgical masks with mask retainers does not differ significantly from surgical masks, except for breathing resistance.

The choice of titanium alloy with 1.2 mm thickness allowed for adequate strength while providing flexibility to achieve better adaptation when pulled towards the mask, and could go through several sterilization methods including pressure steam sterilization. Other advantages of the presented technique include cost-effectiveness and lightweight (approx. 15 g). Titanium has low density (one half of Co-Cr) and high strength, so the mask retainer is light in weight and durable. Meanwhile, the material surfaces can be hollow out to reduce weight and be covered with custom designs that people like (Fig 4). Furthermore, Titanium resources are relatively abundant in China, with large reserves and relatively cheap prices (Dental Titanium powder for printing, $290-$440 per kilogram in China). The cost of the referred titanium mask retainer is about $40 in our study. It can be made not only from titanium, but also from cobalt-chromium, PEEK or resin, and costs around $5 to $40. However, N95 respirators are disposable and the mask retainer can be sterilized and reused, which is sustainable compared to the cost. As smartphones are becoming popular among the public, people may get their 3D face scan data through commercially available face scanning apps at a low price. This study used 3D facial scanning app (Dental Pro; Bellus3D) which no longer offered by the company for new user. Other similar 3D face scanning apps (Heges 3D Scanner, etc.) can be used by people who need them. The data is easily transmitted to the local CAD/CAM center for the design and printing of customized retainers. Anyone only needs a smartphone to gain access to better personal protection for healthcare workers. The mask retainer can be designed online and printed to order online or locally.

In addition to the immediate risk of contracting an epidemic, it has had adverse effects on the mental health of healthcare workers, such as longer working hours, staff shortages, dangerous working environments, and the risk of infecting family members [18]. Healthcare workers in a variety of fields have reported anxiety, distress, depression, and sleep problems [19]. Customized retainers may relieve some physical and psychological pressure on healthcare workers by improving the fitness of masks. It can provide healthcare workers with a personalized protective mask that combines comfort and fit. For healthcare workers, the clinical promotion of the mask retainer is very valuable. Further research is needed to determine whether customized retainers can effectively prevent airborne infections.

## 5. Conclusions

This article presents a digital solution to improve the fit of surgical masks. The workflow incorporated smartphone face scanning and 3D printing techniques. The FF results indicated that using the retainer, the overall FF and each exercise significantly increased compared to that of the surgical mask alone group, and met the fit requirement for KN95 respirators in the Chinese Standard. Meanwhile, compared with N95 respirators, the retainer produces a lower degree of discomfort and is more beneficial to the health of medical staff. It may provide an alternative of personal protective equipment for healthcare workers. Further experiments are required to evaluate the effectiveness of the proposed technique systematically.

## Supporting information

S1 Fig The biometric information of the 10 participants. The biometric information of the 10 participants’ based on the determined landmarks.
(TIF)

S1 Table Fit factor test data. M group: surgical masks without a retainer. MR, group of surgical masks with retainers. iNB, initial normal breathing. DB, deep breathing. Head L/R, head movement from side to side. Head U/D, head movement up and down. fNB, final normal breathing.
(DOCX)

S2 Table Subjective ratings of discomfort in eight domains of 10 subjects. M group: surgical masks without a retainer. MR, group of surgical masks with retainers. The participants were asked at 60, 80 and 100 min of the intermittent exercise how they perceived the comfort in the questionnaire.
(DOCX)
