# Supplementary figures and images for: Development of a customized mask retainer for improving the fit performance of surgical masks
Source: PLoS One. 2022 Dec 9;17(12):e0278889. doi: 10.1371/journal.pone.0278889 (PMC9733890; doi:10.1371/journal.pone.0278889)

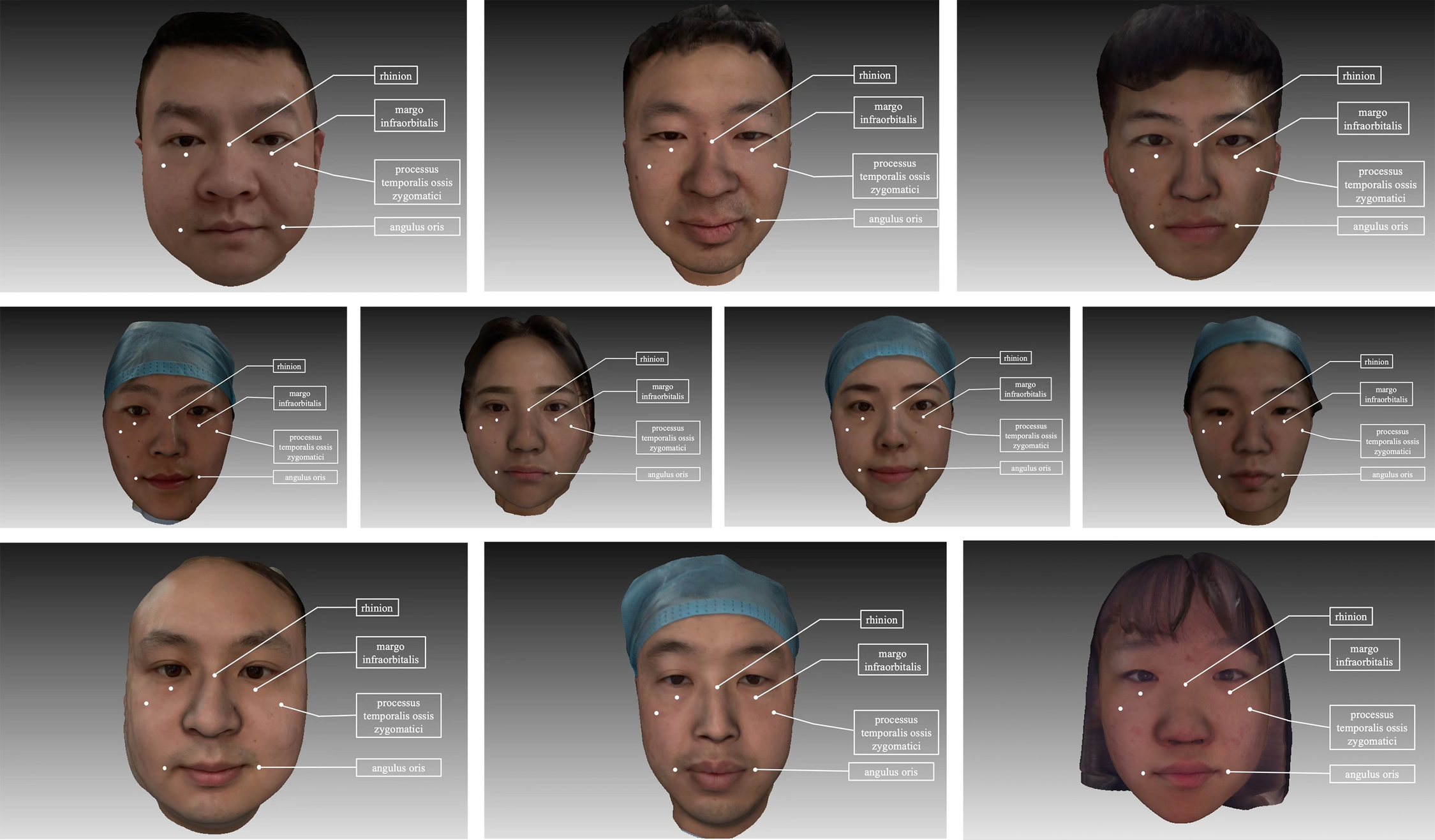

Supplement: S1 Fig — The biometric information of the 10 participants’ based on the determined landmarks. (TIF) [file pone.0278889.s001.tif]
