# Supplementary material for: Development of a customized mask retainer for improving the fit performance of surgical masks
Source: PLoS One. 2022 Dec 9;17(12):e0278889. doi: 10.1371/journal.pone.0278889 (PMC9733890; doi:10.1371/journal.pone.0278889)
Supplement: S1 Table — M group: surgical masks without a retainer. MR, group of surgical masks with retainers. iNB, initial normal breathing. DB, deep breathing. Head L/R, head movement from side to side. Head U/D, head movement up and down. fNB, final normal breathing. (DOCX) [file pone.0278889.s002.docx]

| **Subjects** |  | **A** | **B** | **C** | **D** | **E** | **F** | **G** | **H** | **I** | **J** |
| --- | --- | --- | --- | --- | --- | --- | --- | --- | --- | --- | --- |
| **iNB** | **M** | 4 | 16 | 5 | 11 | 4 | 11 | 17 | 18 | 12 | 9 |
|  | **MR** | 200 | 160 | 200 | 200 | 145 | 200 | 200 | 178 | 200 | 200 |
| **DB** | **M** | 6 | 5 | 4 | 8 | 3 | 6 | 10 | 11 | 6 | 11 |
|  | **MR** | 147 | 123 | 164 | 112 | 141 | 137 | 142 | 146 | 137 | 118 |
| **Head L-R** | **M** | 5 | 8 | 4 | 3 | 7 | 3 | 5 | 4 | 3 | 4 |
|  | **MR** | 148 | 123 | 131 | 146 | 118 | 116 | 125 | 110 | 108 | 110 |
| **Head U-D** | **M** | 6 | 9 | 7 | 7 | 4 | 5 | 16 | 12 | 4 | 5 |
|  | **MR** | 158 | 165 | 128 | 150 | 147 | 129 | 141 | 151 | 128 | 150 |
| **Talk** | **M** | 6 | 5 | 9 | 3 | 6 | 4 | 4 | 9 | 7 | 7 |
|  | **MR** | 51 | 93 | 89 | 78 | 105 | 103 | 79 | 100 | 63 | 80 |
| **fNB** | **M** | 7 | 7 | 15 | 8 | 12 | 4 | 7 | 9 | 11 | 6 |
|  | **MR** | 151 | 171 | 159 | 123 | 200 | 129 | 200 | 155 | 164 | 200 |
| **Overall** | **M** | 5 | 5 | 7 | 6 | 9 | 4 | 5 | 4 | 8 | 6 |
|  | **MR** | 129 | 146 | 150 | 159 | 142 | 168 | 175 | 169 | 146 | 159 |
