# Supplementary material for: Development of a customized mask retainer for improving the fit performance of surgical masks
Source: PLoS One. 2022 Dec 9;17(12):e0278889. doi: 10.1371/journal.pone.0278889 (PMC9733890; doi:10.1371/journal.pone.0278889)
Supplement: S2 Table — M group: surgical masks without a retainer. MR, group of surgical masks with retainers. The participants were asked at 60, 80 and 100 min of the intermittent exercise how they perceived the comfort in the questionnaire. (DOCX) [file pone.0278889.s003.docx]

| **Subjects** |  | **A** | | | **B** | | | **C** | | | **D** | | | **E** | | |
| --- | --- | --- | --- | --- | --- | --- | --- | --- | --- | --- | --- | --- | --- | --- | --- | --- |
| **Breathe resistance** | **M** | 3 | 3.5 | 3 | 2 | 3 | 4.5 | 5 | 5.5 | 6.5 | 4 | 4 | 4 | 6 | 6.5 | 6.5 |
|  | **MR** | 4 | 5 | 7 | 3 | 5 | 7 | 6 | 7 | 8 | 5 | 6.5 | 8 | 6 | 7 | 6.5 |
|  | **N95** | 5.5 | 8.5 | 10 | 4 | 7 | 9 | 7 | 9.5 | 10 | 7 | 8.5 | 10 | 7 | 9 | 9.5 |
| **Itchy** | **M** | 1 | 2 | 1.5 | 4.5 | 5 | 7 | 2 | 4.5 | 6.5 | 2 | 4 | 6 | 2 | 4 | 7 |
|  | **MR** | 0.5 | 1 | 3 | 3 | 4 | 6.5 | 1 | 5 | 6 | 0.5 | 3 | 5 | 3 | 3.5 | 5.5 |
|  | **N95** | 2 | 4 | 6.5 | 4 | 8.5 | 9 | 4.5 | 6 | 7.5 | 3 | 7.5 | 8.5 | 3 | 5 | 7 |
| **Tight** | **M** | 0.5 | 1 | 1 | 2 | 5 | 3 | 2.5 | 3 | 5 | 1 | 3 | 5 | 3.5 | 5 | 5 |
|  | **MR** | 0.5 | 0.5 | 1 | 1 | 1.5 | 1 | 4 | 5.5 | 5 | 0.5 | 1 | 0.5 | 3.5 | 4 | 5.5 |
|  | **N95** | 10 | 9 | 9 | 6 | 8 | 9 | 7 | 7.5 | 9 | 4 | 5 | 4.5 | 5 | 5 | 5.5 |
| **Salty** | **M** | 0 | 1 | 2 | 3 | 2 | 3 | 2.5 | 2 | 2 | 0.5 | 0.5 | 1 | 4 | 4.5 | 5 |
|  | **MR** | 0.5 | 1 | 0.5 | 2 | 2 | 2.5 | 3 | 2 | 2 | 0.5 | 2 | 1.5 | 2.5 | 3 | 3 |
|  | **N95** | 3 | 6 | 7.5 | 4 | 7.5 | 8 | 6 | 6 | 6.5 | 2 | 3.5 | 4 | 5 | 6 | 7.5 |
| **Unfit** | **M** | 0 | 1 | 1 | 3.5 | 4 | 4 | 2 | 2 | 4 | 1 | 1 | 1.5 | 5 | 7 | 7 |
|  | **MR** | 0 | 0.5 | 1 | 4 | 4 | 5 | 0 | 1 | 1 | 4.5 | 5 | 6 | 4 | 5 | 5 |
|  | **N95** | 3 | 4 | 4 | 7 | 8 | 8.5 | 4 | 5 | 5 | 8 | 8 | 10 | 7 | 8.5 | 8 |
| **Odor** | **M** | 0.5 | 1 | 2 | 1.5 | 2 | 2.5 | 1.5 | 3 | 5 | 3 | 2 | 3 | 4 | 5 | 6 |
|  | **MR** | 1 | 3 | 5 | 3.5 | 5 | 5 | 1 | 2 | 2.5 | 1.5 | 2 | 2.5 | 2 | 2 | 2.5 |
|  | **N95** | 6 | 6.5 | 6.5 | 5 | 5.5 | 6 | 3 | 4.5 | 5 | 5.5 | 6 | 6 | 6.5 | 6 | 6 |
| **Fatigue** | **M** | 2 | 4 | 3.5 | 4 | 4.5 | 6 | 1 | 2 | 3 | 1 | 3.5 | 5 | 4 | 7 | 8 |
|  | **MR** | 2.5 | 3.5 | 5 | 5 | 7 | 8 | 2 | 3 | 4 | 2 | 3 | 3.5 | 5 | 7 | 8 |
|  | **N95** | 3 | 4 | 6.5 | 7 | 8.5 | 9 | 4 | 5.5 | 7 | 3 | 5 | 8 | 7 | 8 | 9 |
| **Overall discomfort** | **M** | 4 | 4 | 6 | 5 | 5 | 7 | 5 | 7 | 8 | 3 | 4 | 6.5 | 4 | 6 | 8 |
|  | **MR** | 1 | 3 | 5.5 | 4 | 5 | 8 | 3 | 6.5 | 6 | 5.5 | 6 | 7 | 5.5 | 7 | 9 |
|  | **N95** | 3 | 7 | 8.5 | 4 | 8.5 | 9 | 3 | 6 | 9 | 4 | 8 | 10 | 6 | 6 | 9 |

| **Subjects** |  | **F** | | | **G** | | | **H** | | | **I** | | | **J** | | |
| --- | --- | --- | --- | --- | --- | --- | --- | --- | --- | --- | --- | --- | --- | --- | --- | --- |
| **Breathe resistance** | **M** | 2 | 2.5 | 3 | 3 | 4.5 | 5 | 2 | 5 | 6 | 3.5 | 4.5 | 5 | 4 | 5.5 | 6.5 |
|  | **MR** | 3 | 4 | 5 | 4 | 5 | 6 | 4.5 | 6 | 7 | 5 | 7 | 8.5 | 5 | 6.5 | 8 |
|  | **N95** | 3 | 6.5 | 9.5 | 6 | 9 | 10 | 5 | 7 | 9.5 | 6 | 9 | 9.5 | 6 | 9.5 | 10 |
| **Itchy** | **M** | 1 | 2 | 4 | 0.5 | 2 | 5 | 1 | 2 | 6 | 0.5 | 2 | 4 | 1 | 5 | 6.5 |
|  | **MR** | 2 | 2.5 | 5 | 1.5 | 4 | 5 | 1 | 3 | 5 | 1 | 3 | 5 | 0.5 | 3 | 5 |
|  | **N95** | 2.5 | 5 | 8 | 3 | 4 | 2 | 1.5 | 3 | 3 | 6 | 7 | 9 | 2.5 | 4 | 5 |
| **Tight** | **M** | 6 | 6 | 7 | 4 | 5.5 | 6 | 1 | 2.5 | 2 | 4 | 3 | 5.5 | 1 | 2 | 2 |
|  | **MR** | 5 | 4.5 | 5 | 5 | 4.5 | 5 | 0.5 | 1 | 0.5 | 4 | 5 | 5 | 4 | 3 | 4.5 |
|  | **N95** | 8 | 8.5 | 9 | 7.5 | 7 | 7 | 8 | 9.5 | 9.5 | 5 | 6 | 6.5 | 6 | 5.5 | 6.5 |
| **Salty** | **M** | 0.5 | 1 | 1 | 2.5 | 2 | 3 | 1 | 1.5 | 1.5 | 2.5 | 2.5 | 3 | 2 | 2.5 | 1.5 |
|  | **MR** | 1 | 1 | 1.5 | 2 | 1.5 | 2 | 3 | 2.5 | 2.5 | 2 | 3 | 3 | 0.5 | 1 | 1 |
|  | **N95** | 3 | 5 | 6 | 5 | 7 | 9 | 4 | 6 | 9 | 2 | 4 | 4.5 | 3 | 5 | 7 |
| **Unfit** | **M** | 1 | 4 | 7 | 0 | 1 | 2 | 2 | 5 | 4 | 5 | 5 | 6 | 0.5 | 2 | 5 |
|  | **MR** | 1 | 0.5 | 0.5 | 0 | 0.5 | 0.5 | 2.5 | 2.5 | 2.5 | 4 | 4 | 5 | 0 | 0.5 | 0.5 |
|  | **N95** | 5 | 6.5 | 6.5 | 3 | 4 | 4.5 | 4 | 5 | 6 | 6 | 6.5 | 7 | 3 | 4 | 4 |
| **Odor** | **M** | 1 | 1.5 | 2 | 3 | 5 | 7 | 1 | 1.5 | 2 | 4 | 4.5 | 6 | 1 | 2 | 3 |
|  | **MR** | 1.5 | 2.5 | 2 | 1 | 1.5 | 2 | 2 | 2 | 3 | 6 | 7 | 8 | 3 | 5 | 7 |
|  | **N95** | 3 | 4.5 | 6 | 3.5 | 4 | 5 | 4 | 5.5 | 6 | 6 | 8 | 9 | 4 | 5.5 | 6 |
| **Fatigue** | **M** | 4 | 5 | 5.5 | 3 | 3.5 | 5 | 1 | 2 | 2.5 | 4 | 5 | 7 | 2 | 4.5 | 6 |
|  | **MR** | 3 | 5 | 6 | 2 | 4.5 | 4.5 | 2 | 3 | 5.5 | 5 | 5.5 | 6 | 3 | 4.5 | 5 |
|  | **N95** | 4 | 5 | 6.5 | 5 | 6.5 | 7 | 2 | 3.5 | 5 | 7 | 9 | 10 | 3 | 5 | 8 |
| **Overall discomfort** | **M** | 3 | 3.5 | 7 | 3 | 4 | 5 | 2 | 3.5 | 7 | 5 | 4 | 8 | 1 | 5 | 7 |
|  | **MR** | 2 | 5 | 7 | 3 | 5 | 7 | 3 | 4.5 | 7 | 4.5 | 7 | 9 | 3.5 | 5 | 8 |
|  | **N95** | 4 | 6 | 7 | 5 | 6.5 | 8 | 5 | 6 | 8.5 | 6 | 9 | 10 | 6 | 8.5 | 10 |
